# Fast cortical surface reconstruction from MRI using deep learning

**DOI:** 10.1186/s40708-022-00155-7

**Published:** 2022-03-09

**Authors:** Jianxun Ren, Qingyu Hu, Weiwei Wang, Wei Zhang, Catherine S. Hubbard, Pingjia Zhang, Ning An, Ying Zhou, Louisa Dahmani, Danhong Wang, Xiaoxuan Fu, Zhenyu Sun, Yezhe Wang, Ruiqi Wang, Luming Li, Hesheng Liu

**Affiliations:** 1 National Engineering Laboratory for Neuromodulation, School of Aerospace Engineering, Tsinghua University, Beijing, 100084 China; 2 Athinoula A. Martinos Center for Biomedical Imaging, Department of Radiology, Massachusetts General Hospital, Harvard Medical School, Charlestown, MA 02129 USA; 3 School of Computer Science and Technology, University of Science and Technology of China, Hefei, 230027 China; 4 Neural Galaxy, Beijing, 102206 China; 5 Academy for Advanced Interdisciplinary Studies, Peking University, Beijing, 100080 China; 6 Department of Neuroscience, Medical University of South Carolina, Charleston, SC 29425 USA; 7 State Key Laboratory of Reliability and Intelligence of Electrical Equipment, Hebei University of Technology, Tianjin, 300401 China; 8 Precision Medicine and Healthcare Research Center, Tsinghua-Berkeley Shenzhen Institute, Tsinghua University, Shenzhen, 518055 China; 9 IDG/McGovern Institute for Brain Research at Tsinghua University, Beijing, 100084 China; 10 Beijing Neurosurgical Institute, Capital Medical University, Beijing, China

**Keywords:** Cortical surface reconstruction, Level set, Deep learning, T1-weighted MRI, FreeSurfer

## Abstract

Reconstructing cortical surfaces from structural magnetic resonance imaging (MRI) is a prerequisite for surface-based functional and anatomical image analyses. Conventional algorithms for cortical surface reconstruction are computationally inefficient and typically take several hours for each subject, causing a bottleneck in applications when a fast turnaround time is needed. To address this challenge, we propose a fast cortical surface reconstruction (FastCSR) pipeline by leveraging deep machine learning. We trained our model to learn an implicit representation of the cortical surface in volumetric space, termed the “level set representation”. A fast volumetric topology correction method and a topology-preserving surface mesh extraction procedure were employed to reconstruct the cortical surface based on the level set representation. Using 1-mm isotropic T1-weighted images, the FastCSR pipeline was able to reconstruct a subject’s cortical surfaces within 5 min with comparable surface quality, which is approximately 47 times faster than the traditional FreeSurfer pipeline. The advantage of FastCSR becomes even more apparent when processing high-resolution images. Importantly, the model demonstrated good generalizability in previously unseen data and showed high test–retest reliability in cortical morphometrics and anatomical parcellations. Finally, FastCSR was robust to images with compromised quality or with distortions caused by lesions. This fast and robust pipeline for cortical surface reconstruction may facilitate large-scale neuroimaging studies and has potential in clinical applications wherein brain images may be compromised.

**Supplementary Information:**

The online version contains supplementary material available at 10.1186/s40708-022-00155-7.

## Introduction

The human cerebral cortex can be viewed as a 2-dimensional surface sheet folded in 3-dimensional (3D) space with highly individualized organizational and topological patterns [1–4]. Reconstructing cortical surfaces from magnetic resonance imaging (MRI) not only facilitates the quantification of brain morphometric measures, such as cortical thickness [4, 5] and sulcal depth [6, 7], but also allows for visualization of surface-based renderings displaying a variety of functional and anatomical measures [8]. Surface-based image analyses have shown advantages over traditional volume-based approaches in aligning imaging data from different subjects [9] and have been widely adopted in both basic and clinical neuroscience research [10–16]. For these reasons, automatic cortical surface reconstruction has become a critical step in the processing pipelines for analyses of data derived from functional MRI (fMRI) [17], magnetoencephalography (MEG) [18], and electroencephalography (EEG) [19] techniques. However, current cortical surface reconstruction (CSR) algorithms are computationally inefficient, restricting their use in some scenarios where fast processing is desired [3, 20–23]. For example, reconstructing a single subject’s brain surface using the FreeSurfer pipeline typically takes several hours [24, 25]. The high computational cost is mainly driven by traditional computer vision algorithms and their dependency on multi-step preprocessing. Recently, advances in machine learning have offered opportunities to accelerate brain image processing. For example, Henschel et al. proposed a “FastSurfer” pipeline to speed up anatomical segmentation using deep learning [26] and Cheng et al. proposed a “SphereMorph” algorithm to accelerate surface registration [27]. Cruz and coworkers proposed a “DeepCSR” algorithm to reconstruct cortical surfaces using deep learning, which is capable of capturing precise cortical details but does not significantly shorten the processing time [25]. To date, an efficient approach for rapid reconstruction of brain surfaces within a few minutes is still lacking.

Here, we propose a novel, fast cortical surface reconstruction (FastCSR) algorithm based on deep learning. We trained a 3D U-Net model to learn implicit representations of cortical surfaces from original T1-weighted (T1w) images and then reconstructed topology-preserving cortical surface meshes from these representations. In contrast to explicit surface representation based on triangular meshes, the implicit cortical surface representation, termed level set [28], reconstructs the cortical surface in volumetric space in an implicit manner. Specifically, in the level set representation, voxels whose values equal zero represent surface boundaries, whereas a negative or positive value of a voxel represents the distance from the surface boundaries inward or outward, respectively. Once the level set representations are learned from the inputted anatomical T1w images, the explicit surface meshes can be easily generated by extracting zero values in the representations without topological defects [29]. Herein, we aim to accelerate CSR while at the same time maintaining compatibility with the FreeSurfer pipeline, the latter of which represents one of the most widely used automatic CSR pipelines currently available [7]. Therefore, we trained a supervised model using level set representations of cortical surfaces reconstructed from FreeSurfer. Our FastCSR approach is able to reconstruct an individual’s brain surface within 5 min. In addition to quantifying differences in processing time, we also compared the displacement and morphometrics of reconstructed surfaces using our method compared to two commonly employed pipelines (i.e., FreeSurfer and FastSurfer) using multiple datasets that varied in their scanning protocols and acquisition parameters, as well as image quality. Finally, we examined the generalizability of our deep learning method in previously unseen datasets, and applied this method to reconstruct surfaces for brains with distortions caused by lesions.

## Materials and methods

### Datasets

Multiple datasets were employed in the current study. To train and validate the FastCSR model, we used publicly available T1w images of 808 subjects from the Consortium for Reliability and Reproducibility (CoRR) and the Southwest University Adult Lifespan Dataset (SALD). Test–retest reliability was evaluated using a separate CoRR dataset collected in the Hangzhou Normal University (CoRR-HNU). To test the generalizability of our method in previously unseen datasets, we utilized data of 30 subjects from the Human Connectome Project Young Adult (HCP) dataset and 30 autistic patients from the Autism Brain Imaging Data Exchange II (ABIDE-II) dataset. Furthermore, we examined the performance of our method in a few patients whose brains were distorted due to cerebral stroke (i.e., Stroke dataset). These datasets are described in detail below and demographics, usages, sources, clinical states, and scanners are summarized in the Additional file 1: Table S1.

#### CoRR datasets

To train and validate our method, we used 11 datasets from CoRR, which included 331 healthy participants (ranging from 6 to 62 years of age). These datasets were collected on different 3 T scanners, including Siemens TrioTim (Siemens Healthcare), GE Signa HDxt, and GE Discovery MR750 (GE Healthcare System) at voxel resolutions varying from 0.9 to 1.3 mm. These datasets are listed in the Additional file 1: Table S1. Detailed descriptions of scanning protocols and demographics can be found in the previous report [30] and the website (https://fcon_1000.projects.nitrc.org/indi/CoRR/html/index.html).

#### CoRR-HNU dataset

To evaluate test–retest reliability, we used the CoRR-HNU dataset which consists of 30 healthy adults, each of whom underwent 10 scanning sessions across a one-month period [30]. Of note, the CoRR-HNU dataset was not part of the 11 CoRR datasets described above. The T1w images were acquired on a GE Discovery MR750 3 T scanner equipped with an 8-channel head coil using a 3D “spoiled-gradient-echo” (SPGR) sequence (TR = 8.06 ms, TE = min full, TI = 450 ms, flip angle = 8°, field of view (FOV) = 250 × 250, voxel size = 1 × 1 × 1 mm^3^). The study was approved by the Ethics Committee of the Center for Cognition and Brain Disorders at Hangzhou Normal University and written informed consent was obtained from each participant before commencement of any experimental procedures.

#### SALD dataset

We used all 494 subjects from the SALD dataset (307 women; 187 men; 19–80 years old). Participants’ T1w images were acquired on a Siemens TrioTim 3 T scanner using a magnetization-prepared rapid gradient echo (MPRAGE) sequence (repetition time (TR) = 1.9 s, echo time (TE) = 2.52 ms, flip angle = 90°, voxel size = 1 × 1 × 1 mm^3^). This study was approved by the Research Ethics Committee of the Brain Imaging Center of Southwest University. Written informed consent was obtained from each participant prior to the start of the study.

#### HCP dataset

We randomly sampled 30 young healthy participants from HCP S900 release (15 females; 15 males; 22–35 years old). Their T1w images were acquired with a Siemens Connectome Skyra 3 T scanner using a high-resolution MPRAGE sequence (TR = 2.4 s, TE = 2.14 ms, TI = 1 s, flip angle = 8°, FOV = 224 × 224, voxel size = 0.7 × 0.7 × 0.7 mm^3^). The local review board at Washington University in St. Louis approved all study procedures. Written informed consent was obtained from each participant before study enrollment.

#### ABIDE-II dataset

The ABIDE-II project consists of 521 autistic patients from 19 different sites [31] (http://fcon_1000.projects.nitrc.org/indi/abide/abide_II.html). We used stratified sampling to select 30 patients from the full dataset. The sampling data were acquired using a Philips Achieva 1.5 T scanner and various other 3 T scanners, including the GE MR750, Siemens TriTim, Allegra, and Skyra scanners, and a Philips Ingenia scanner. The spatial resolutions of sampled data varied from 0.7 to 1.3 mm. Prior to data collection, all sites were required to confirm that their local Institutional Review Board (IRB) or ethics committee have approved study procedures. Scanning protocols and demographics of this dataset were described in a previous report published by [31].

#### Stroke dataset

We examined our FastCSR method on three stroke patients whose brains were distorted due to severe cerebral hemorrhage. Their T1w images were acquired on a Philips Ingenia 3 T scanner (Philips Healthcare, Best) with a sagittal 3D T1w sequence (TR = 1000 ms, TE = 2.15 ms, flip angle = 8°, FOV = 256 × 256, voxel size = 1 × 1 × 1 mm^3^) at the China Rehabilitation Research Center, Beijing, China. Written informed consent was obtained from each participant in accordance with guidelines and regulations previously approved by the Medical Ethics Committee of China Rehabilitation Research Center.

### FastCSR pipeline

**Fig. 1:**
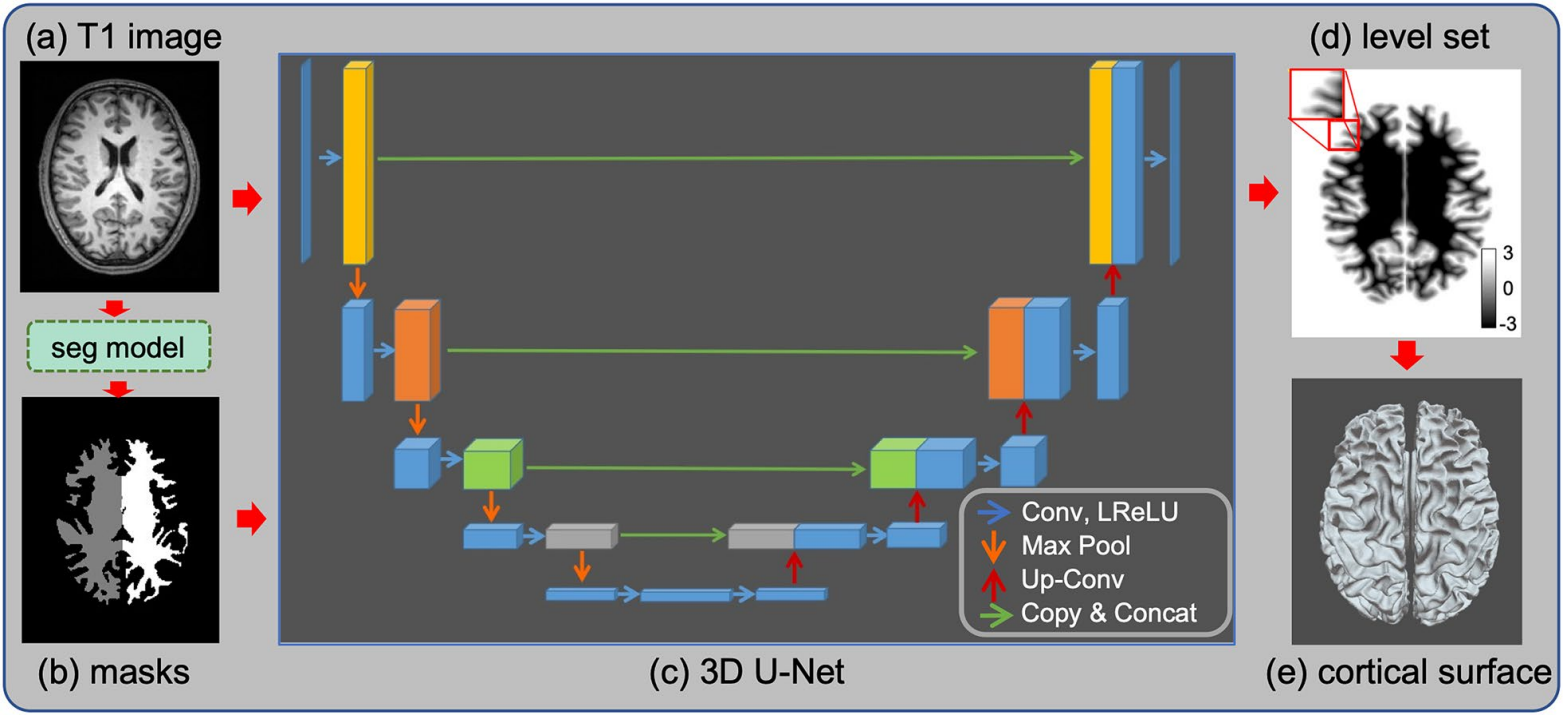
Schematic of the fast cortical surface reconstruction (FastCSR) workflow. The FastCSR workflow can be summarized in four steps: **a** original T1w images are normalized and fed to a 3D U-Net for segmentation of white matter from gray matter. **b** After segmentation, hemispheric white matter masks are generated, distinguishing the two hemispheres in the T1w images. **c** The hemispheric masks and the original T1w images are fed to another 3D U-Net to predict the level set representation of the cortical surface. Level set is an implicit representation of the cortical surface. The U-shaped network architecture can be briefly described as follows: each blue arrow represents a 3 × 3 × 3 convolution process followed by a leaky rectified linear unit (LReLU). Each orange downward arrow indicates a 2 × 2 × 2 max pooling. Each red upward arrow indicates a 2 × 2 × 2 upsampling followed by a convolution (Up-Conv). The long-range skip connections using the copy-and-concatenate (Copy & Concat) operation are indicated by green arrows. **d** The level set representations of the surfaces are generated by the deep learning model. The voxels whose level set value equals to zero delineate the boundary of the cortical surface. Negative voxels indicated by dark colors are below the surface and positive voxels indicated by light colors are above the surface. The red box shows the level set representation in the left frontal cortex magnified to better visualize the details in the surface boundary. **e** An explicit surface mesh is reconstructed from the level set representation through a fast topology-preserving isosurface extraction algorithm. The resulting surface is visualized using a dorsal orientation

Our FastCSR pipeline for cortical surface reconstruction is summarized as follows (see Fig. 1): first, intensities of original T1w images are normalized to 0–255 and then z-scored. Second, the normalized images are fed to a 3D U-Net for segmentation and then white matter masks of both hemispheres are generated (Fig. 1a, b). The masks provide white matter priors separately for left and right hemispheres in the following level set regression. Third, the white matter masks and the normalized T1w images are fed to a 3D U-Net, which was adapted for regression to learn the level set representation (Fig. 1c). Finally, a topology-preserving surface extraction method is applied (Fig. 1d) to obtain the explicit surface mesh representations (Fig. 1e).

#### A uniform 3D U-Net architecture for both segmentation and regression

To achieve accurate anatomical segmentation and level set regression from the 3D T1w images, we applied a 3D U-Net framework since this approach integrates features at different scales and captures both complex and fine-grained surface boundaries, as well as global features. Here, we adopted a state-of-the-art 3D U-Net network architecture, using the no-new-U-Net (nnU-Net) framework [32]. The nnU-Net was designed to efficiently perform automatic configuration for arbitrary new datasets and can achieve high performance for a variety of segmentation tasks [32–34].

As shown in Fig. 1c, the nnU-Net architecture closely resembles the original U-Net [35] and its 3D counterpart [36] with a contracting path (the left arm) and an expansive path (the right arm). In the contracting steps, each layer consists of a 3 × 3 × 3 convolution followed by a leaky rectified linear unit (LReLU), and then a 2 × 2 × 2 max pooling with strides of two in each dimension for downsampling. In the expansive steps, each layer consists of an up-convolution of 2 × 2 × 2 with strides of two in each dimension for upsampling, and then a 3 × 3 × 3 convolution followed by a LReLU. The shortcut connections copy layers with equal resolution in the contracting path and concatenate layers in the expansive path to add essential high-resolution features during upsampling processing.

For the segmentation of the hemispheric white matter masks, benefiting from the automatic configuration, we directly applied this framework without manual tuning architectures or parameters. For the level set regression, we adapted the uniform 3D U-Net architecture and modified the output layer and the loss function for the regression task. Specifically, we replaced the output layer from a softmax layer to a convolutional layer and used the mean squared error (MSE) instead of cross-entropy as the loss function. Moreover, to avoid loss of precision in the regression task, we turned off data enhancement parameters, such as scaling, rotation, and flipping. In sum, the 3D U-Net architecture consisted of 116 layers, including 30,785,248 trainable parameters.

#### Surface extraction and optimization

To efficiently extract surface meshes from the implicit surface representation, we used a fast volumetric topology correction method and a topology-preserving surface extraction method, packaged in the Nighres toolbox [29]. The volumetric topology correction method leveraged an efficient propagation algorithm using all available information from level set representation [37] and a topology-preserving geometric deformable surface model to extract the surface mesh from the level set [38]. A surface optimization method from FreeSurfer (see command mris_make_surfaces) could be optionally added to further improve the surface reconstruction accuracy.

### Model training

#### Training data and labels

For model training, we used stratified sampling to select 80% of subjects (*n* = 646) from the CoRR and the SALD datasets. The remaining subjects (*n* = 162) comprised the validation dataset. All training data were processed using FreeSurfer v6.0 release [7, 39] (http://surfer.nmr.mgh.harvard.edu/). To train the segmentation model, we generated white matter mask labels based on the anatomical segmentation from FreeSurfer. To train the level set representation learning model, we obtained the training labels of level set representations by estimating the voxel-wise distance from the surfaces reconstructed by FreeSurfer, using the FreeSurfer command mris_volmask. Throughout the transformation, the explicit surfaces were embedded in the implicit surface representations.

#### Network implementation

The network model was implemented in Python 3.8.8 using PyTorch framework 1.6.0 [40]. The model was trained using an Adam optimizer [41] with a batch size of 2, a patch size of 128 × 160 × 112, and an initial learning rate of 0.001 that decreased by 10^–6^ for each epoch. The maximal number of training epochs was set to 1000 and training was terminated once the loss function converged in 50 consecutive epochs. We trained the model in a hardware environment that consisted of an Intel Xeon Platinum 8259CL CPU and a Nvidia Tesla T4 GPU with 16 GB RAM. The training time of the model was 127.2 h.

### Model evaluations

We compared our FastCSR method with the “recon-all” pipeline from the FreeSurfer v6.0 release (https://surfer.nmr.mgh.harvard.edu) and the “recon-surf” pipeline from the FastSurfer (https://github.com/Deep-MI/FastSurfer). To evaluate the performance of these different methods compared to our FastCSR pipeline, we examined the processing time, mesh quality and surface displacement. Moreover, we evaluated the generalizability of methods in unseen datasets by examining anatomical cortical parcellations, morphometrics (i.e., cortical thickness, sulcal depth), and gray–white contrast. Finally, we evaluated the test–retest reliability of the aforementioned morphometrics and anatomical parcellations in a multi-session dataset.

#### Processing time

The processing time was estimated in the aforementioned hardware environment. We assessed processing time of both sequential and parallel processing pipelines. The parallel processing indicates parallelization of two hemispheres. For both sequential and parallel processing, we assessed processing time for the surface reconstruction step and for the whole pipeline. For the surface reconstruction step, the inputs contained the preprocessed T1w images and necessary files and the outputs were explicit mesh representations. The whole pipeline consisted of the necessary preprocessing steps and the surface reconstruction step. The processing time was averaged across subjects. The coefficient of variation in the processing time (CV_PT_) was defined as ratio of the standard deviations in processing time across subjects and the mean processing time across subjects. Lower CV value represented greater stability and certainty in processing time.

#### Mesh quality

The mesh quality was calculated by taking the average of triangle qualities across all triangles in the cortical meshes. The triangle quality was estimated by

$$Q=\frac{4\sqrt{3}A}{{e}_{1 }^{2}+ {e}_{2 }^{2}+ {e}_{3 }^{2}},$$

where *A* is the area of a triangle and *e*_*i*_ are the edges of the triangle [42]. A *Q* value equivalent to 1 represents an equilateral triangle with high mesh quality, whereas a *Q* of zero represents a degenerated triangle, indicating low mesh quality.

#### Surface displacement

Surface displacement was estimated for each of the three different methods (i.e., FastCSR, FastSurfer or FreeSurfer) by calculating the distance between the surface reconstructed at each vertex [24]. A smaller displacement indicated greater similarity between the two surfaces. We projected the vertex-wise displacement map to a common FreeSurfer surface space (i.e., fsaverage6) and averaged the vertex-wise displacement values across subjects in the validation set [43].

#### Morphometrics

We also evaluated two commonly used morphometrics, cortical thickness and sulcal depth. Cortical thickness measures the thickness of the cortical gray matter and was estimated by calculating the shortest distance between white matter and pial surfaces at each vertex [4, 44]. Sulcal depth measures how far a vertex is from a hypothetical ‘midsurface’ point between the gyrus and sulcus [6]. A positive value indicated sulcus and a negative value indicated gyrus [45].

#### Dice coefficients of anatomical cortical parcellations

In the reconstructed surfaces, 34 anatomical regions of interest (ROIs) were automatically identified according to cortical neuroanatomical features using FreeSurfer [46, 47]. Spatial similarity of anatomical ROIs derived from FreeSurfer and FastCSR were compared using Dice coefficient.

### Test–retest reliability

For the aforementioned morphometrics, instability was estimated as the standard deviations obtained from the vertex-wise morphometrics across 10 repeated scans of each subject. For the anatomical parcellation, instability was estimated as the standard deviations in the Dice coefficients of anatomical ROIs between the session-level parcellation and the session-averaged parcellation across the 10 scan sessions for each subject. The instability values were averaged across all 30 subjects. Lower instability was indicative of higher test–retest reliability in the surface reconstruction.

### Statistical analyses

To test whether the surface displacements between FastCSR and FreeSurfer were different from displacement between FastSurfer and FreeSurfer, we used the two-sample Kolmogorov–Smirnov test between the two distributions. Similarly, distribution differences for instability of morphometrics and anatomical parcellations between FastCSR and FreeSurfer were also compared using two-sample Kolmogorov–Smirnov tests. Two-tailed paired samples *t*-tests were applied to compare the mesh quality between FreeCSR and FreeSurfer as well as to compare the resulting morphometrics and anatomical parcellations obtained from the FastCSR versus FreeSurfer pipelines. For all statistical tests, multiple comparisons were corrected using false discovery rate (FDR).

## Results

### FastCSR efficiently reconstructs brain surfaces

**Fig. 2:**
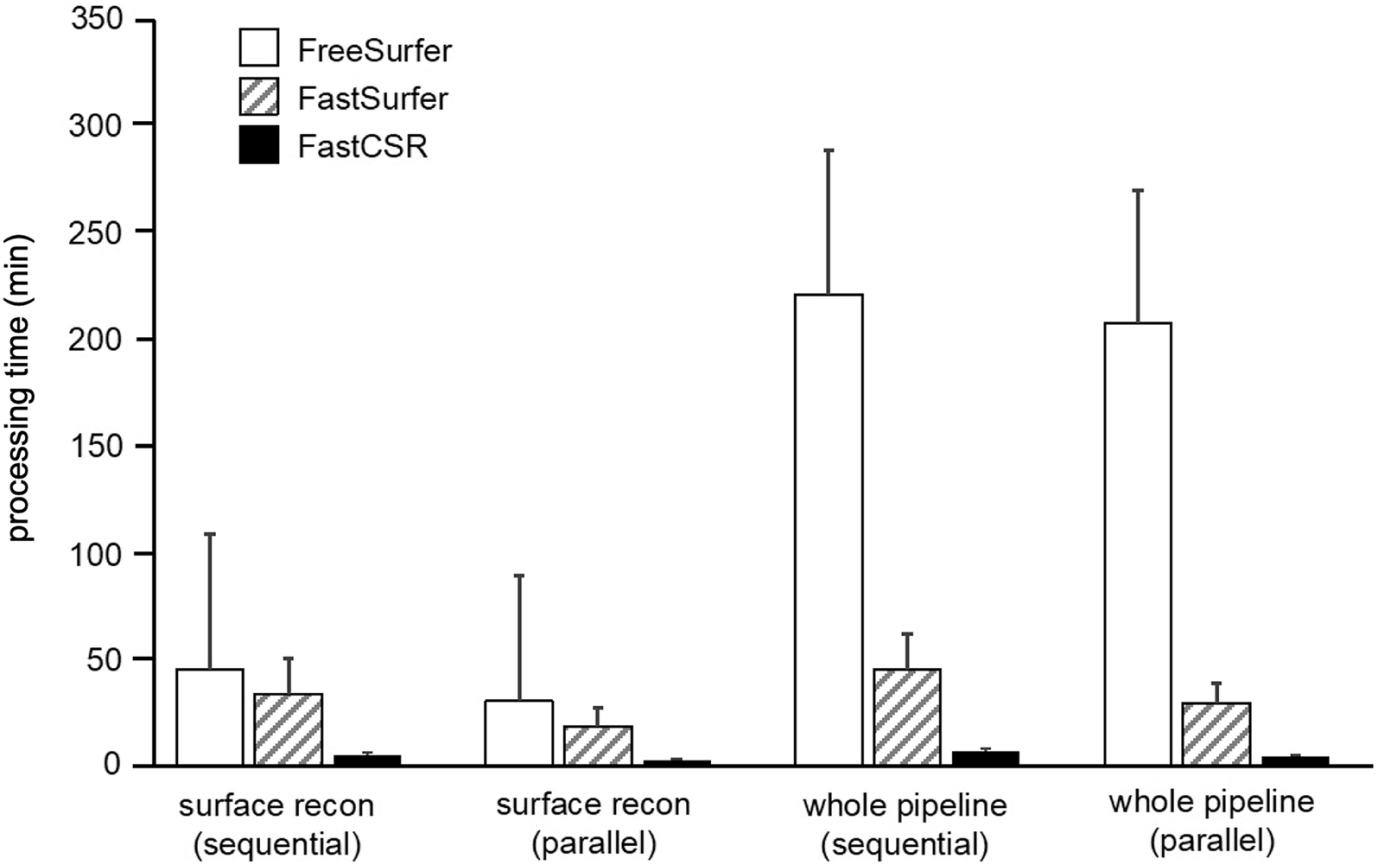
Processing time of FastCSR, FreeSurfer and FastSurfer. The bar graphs illustrate differences in the average processing time for the validation dataset using FreeSurfer, FastSurfer and FastCSR. We compared the processing times for the surface reconstruction step alone (surface recon) and the whole pipeline including all necessary preprocessing steps for surface reconstruction using either sequential or parallel processing, the latter of which processes both hemispheres simultaneously. Among the three methods, FastCSR achieved the highest computational efficiency for the surface reconstruction step (5.22 ± 0.92 min and 2.61 ± 0.46 min for sequential and parallel processing, respectively), as well as for the whole pipeline (7.05 ± 0.92 min and 4.44 ± 0.46 min for sequential and parallel processing, respectively). Error bars indicate standard deviations

To compare the computational efficiency of FreeSurfer, FastSurfer, and FastCSR, we employed these three methods to reconstruct brain surfaces using T1w images with 1.0-mm isotropic resolution in the validation dataset (*n* = 162). Processing times for each method were evaluated in the same hardware environment (Fig. 2). The sequential FreeSurfer and FastSurfer pipelines took 45.06 ± 63.86 min (mean ± std.) and 33.64 ± 16.42 min to complete the surface reconstruction step (surface recon), respectively, while our FastCSR model accomplished this task in 5.22 ± 0.92 min. Since our FastCSR method can process both hemispheres in parallel, the FastCSR pipeline further reduced the processing time to 2.61 ± 0.46 min. The stability of processing time across methods was evaluated by CV_PT_. The CV_PT_ of FreeSurfer, FastSurfer, and FastCSR were 1.42, 0.49, and 0.17, respectively. The higher CV_PT_ obtained with FreeSurfer and FastSurfer pipelines indicated greater variability in processing times resulting from differing numbers of initial topology defects encountered. In contrast, the processing time of FastCSR was highly stable and could be reliably estimated.

Computational time for the whole pipeline, which included multiple preprocessing steps in addition to the surface reconstruction step, was also examined. Our parallel FastCSR method completed the whole pipeline in 4.44 ± 0.46 min, approximately 47 times faster than FreeSurfer (207.19 ± 62.22 min) and about 7 times faster than the parallel FastSurfer method (29.59 ± 9.08 min).

Furthermore, we evaluated the extent to which processing time is influenced by the spatial resolution of the T1w images. We recorded processing times for the surface reconstruction step using FreeSurfer and our FastCSR method with high-resolution T1w images (0.7-mm isotropic) obtained from the HCP dataset. FreeSurfer took almost 17 times longer to process the high-resolution 0.7-mm isotropic T1w images (507.32 ± 470.62 min) compared to the lower resolution T1w 1.0-mm isotropic images (30.26 ± 59.15 min). In contrast to FreeSurfer, our FastCSR approach took about 1.5 times longer to process the higher resolution 0.7-mm isotropic images (3.93 ± 0.06 min) relative to the lower resolution 1.0-mm isotropic images (2.61 ± 0.46 min), indicating that FastCSR is particularly well-suited for processing submillimeter-resolution images in a temporally efficient manner.

### FastCSR reconstructs brain surfaces with high mesh quality

**Fig. 3:**
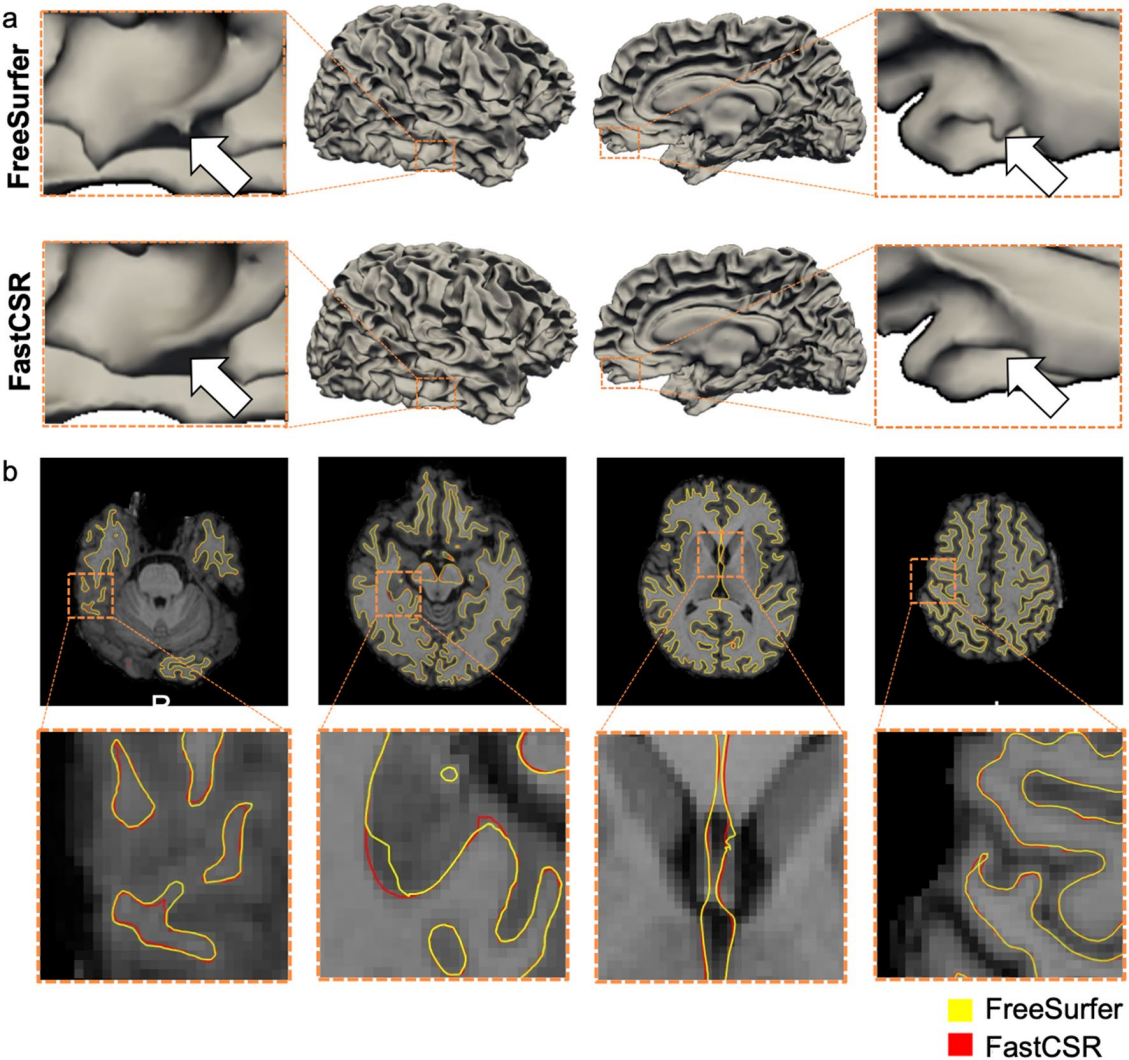
Surfaces reconstructed by FastCSR are comparable to results from FreeSurfer. **a** The cortical surfaces from a randomly selected healthy participant reconstructed using the FreeSurfer (the upper panel) and the FastCSR (the lower panel) pipelines. The surfaces generated by different methods show high similarity with small discrepancies that are highlighted by orange dashed boxes. **b** Horizontal slices at multiple levels taken from the participant’s T1w image showing the cortical surface identified using FreeSurfer (yellow lines) and FastCSR (red lines) show that both methods accurately capture the boundary between white and gray matter. The surfaces derived from these two methods showed a high degree of overlap in most of cortical areas, indicating high concordance between these two methods. Small discrepancies are highlighted by orange dashed boxes

**Fig. 4:**
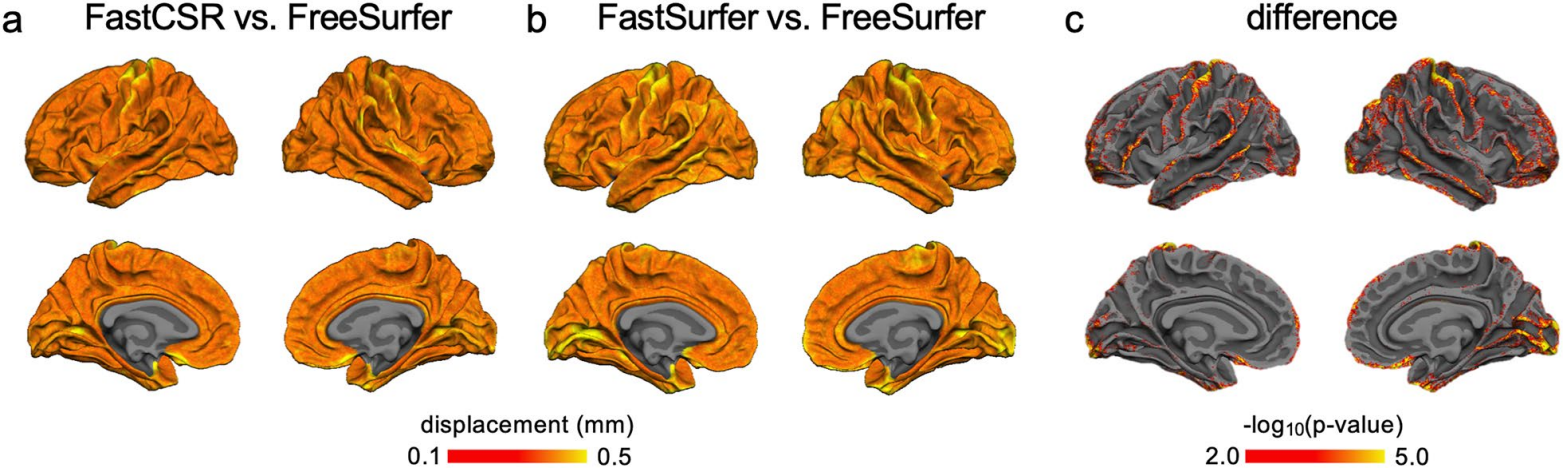
Differences in surfaces reconstructed by FastCSR, FreeSurfer and FastSurfer. **a** To quantitatively assess the surface agreement, we measured the average surface displacement between FastCSR and FreeSurfer across participants in the validation set. The maximal displacement between FreeSurfer and FastCSR was smaller than 0.5 mm, which is approximately half of the voxel size. **b** The average surface displacement between FastSurfer and FreeSurfer was also measured. The average surface displacement map showed a similar pattern with that observed from using the FastCSR method (Spearman’s *ρ* = 0.714, p < 0.0001). **c** The direct comparison of displacement maps between the FastCSR vs. FreeSurfer contrast and the FastSurfer vs. FreeSurfer contrast was performed. Results showed that the FastCSR approach achieved overall better performance. Lateral gyri and visual cortices showed significantly smaller displacement in the FastCSR versus FreeSurfer method than FastSurfer versus FreeSurfer (two-tailed paired *t*-tests, *p* < 0.01, FDR corrected)

Given that FreeSurfer is the most widely used pipeline for brain surface reconstruction, we compared the surfaces reconstructed by FastCSR and FreeSurfer. Geometrical patterns of surfaces reconstructed by these two pipelines were highly similar (see Fig. 3a for results of a randomly selected subject). Likewise, the surface boundaries from these two pipelines also showed high degree of overlap (see Fig. 3b for a randomly selected subject). To quantitatively assess the similarity of surfaces derived from different pipelines, we measured the average displacement between FastCSR and FreeSurfer surface across participants in the validation dataset. As a reference, we also compared the displacement between FastSurfer and FreeSurfer results (Fig. 4). The displacement between FastCSR and FreeSurfer results was mainly observed near the orbitofrontal cortex (OFC), calcarine fissure, and pre- and post-central gyri. However, the average displacement was diminutive in comparison, and the maximum displacement was less than 0.5 mm, which is about half of the voxel size (Fig. 4a). The displacement between FastSurfer and FreeSurfer showed a similar pattern (Spearman’s *ρ* = 0.71, *p* < 0.001; Fig. 4b), whereas the displacement of FastSurfer was significantly greater than that obtained using the FastCSR mainly in lateral gyri and visual cortices (Fig. 4c; two-tailed paired *t*-tests, *p* < 0.01, FDR corrected).

**Fig. 5:**
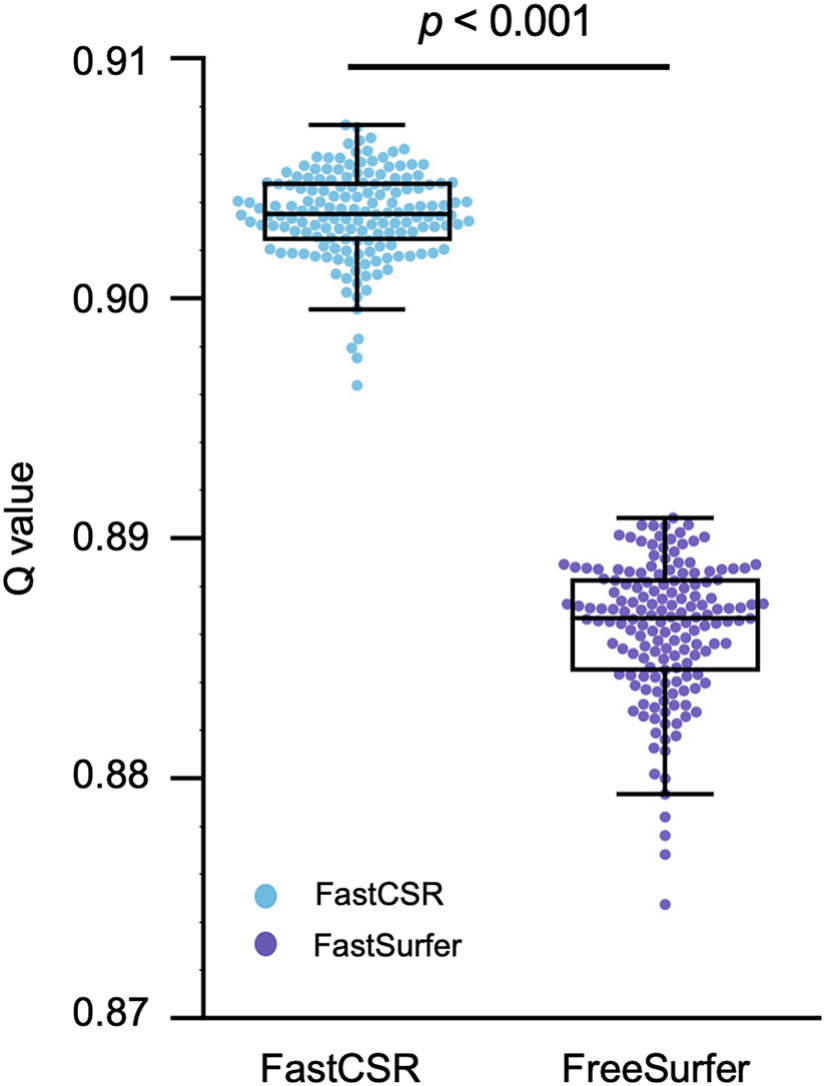
Surfaces reconstructed by FastCSR and FreeSurfer show comparable mesh quality. To assess the mesh quality of the FastCSR surface, we estimated the mesh quality in terms of *Q* value in the validation set. The *Q* values of FastCSR (blue dots) and FreeSurfer (purple dots) are illustrated using swarm plots, with the means of each distribution depicted by boxplots with boxes marks the high and low quartiles and whiskers indicating the minimum and maximum values. The *Q* values obtained with FastCSR (*Q*_FastCSR_ = 0.903 ± 0.002) are significantly higher than those of FreeSurfer (*Q*_FreeSurfer_ = 0.886 ± 0.003; two-tailed paired *t*-test, *t*_(161)_ = 122.008, *p* = 1.462 × 10^–160^), indicating greater mesh quality achieved with our approach

To compare the quality of cortical surface meshes obtained using the three different pipelines, we estimated the *Q* value of each mesh triangle, which indicates the uniformity of the triangle [26]. In the validation dataset, surfaces reconstructed by FastCSR showed high *Q* values (*Q*_FastCSR_ = 0.903 ± 0.002, mean ± std.), indicating good mesh quality with triangles close to equilateral. Surfaces reconstructed by FreeSurfer showed slightly lower mesh quality (Fig. 5; *Q*_FreeSurfer_ = 0.899 ± 0.003; two-tailed paired *t*-test, *t*_(161)_ = 122.01, *p* = 1.46 × 10^–160^).

### FastCSR has good generalizability in previously unseen data

Given that FastCSR is based on deep machine learning, which inherently relies on a training dataset, an important question is whether the trained model can be generalized to unseen datasets with different sample distributions from the training set. To examine the generalizability of FastCSR, we employed the ABIDE-II dataset which consists of 30 autism spectrum disorder (ASD) patients [31] and the HCP dataset which consists of 30 healthy participants [48]. The ABIDE-II dataset was collected from 19 different centers utilizing different MR platforms (GE, Siemens, and Philips), spatial resolutions (from 0.7 to 1.3 mm), and magnetic field strengths (1.5 T and 3.0 T). The spatial resolution of T1w images of the HCP dataset is 0.7 mm isotropic, which is higher than the resolutions of our training data (1.0 mm isotropic). In these two test datasets, we first reconstructed the surfaces using the FreeSurfer pipeline as a reference, which does not rely on training.

**Fig. 6:**
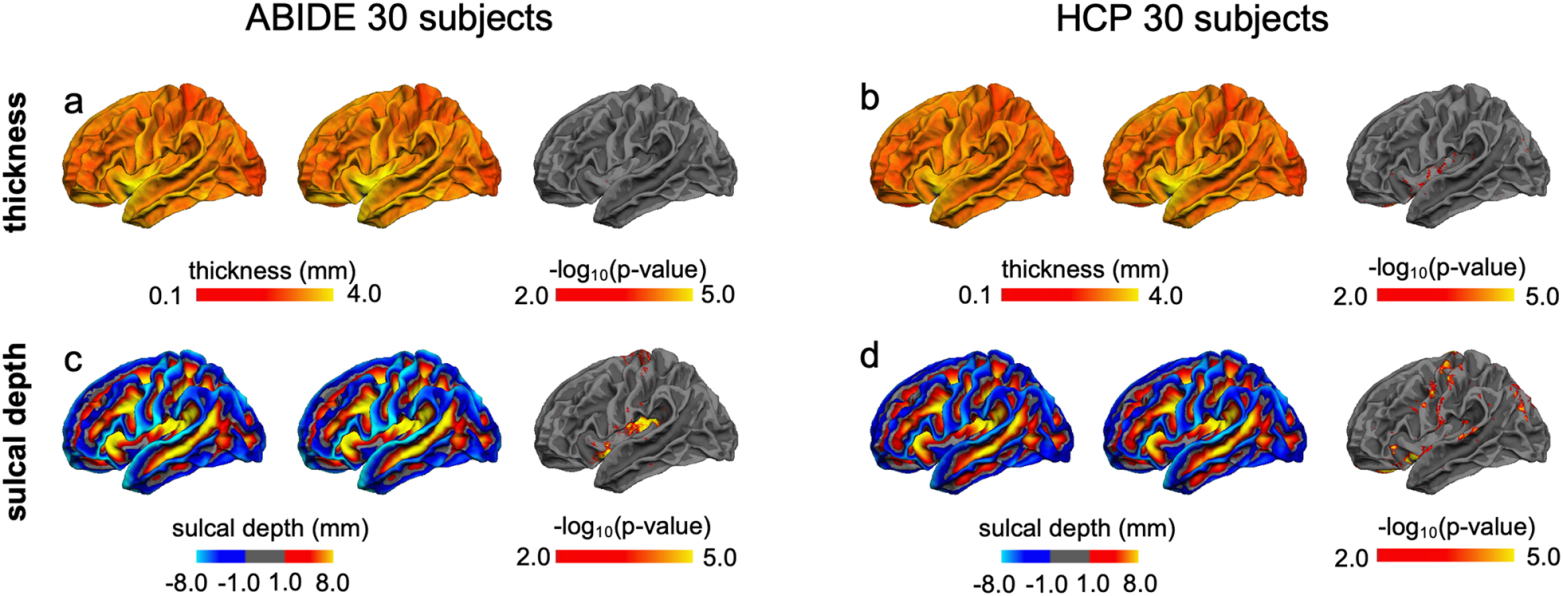
Surface morphometries measured in unseen datasets. To examine if FastCSR is generalizable to unseen datasets, we applied this method to the previously unseen ABIDE-II dataset with T1w images at 1.0-mm resolution and the HCP dataset with 0.7-mm resolution images. These data were also processed using the FreeSurfer pipeline. Cortical thickness and sulcal depth were estimated. **a** The average cortical thickness maps obtained from FreeSurfer (left) versus FastCSR (right) in the ABIDE-II dataset are similar, with only 0.07% of the vertices demonstrating significant difference (two-tailed paired *t*-tests, *p* < 0.01, FDR corrected). **b** For the HCP dataset, the average cortical thickness maps derived from FreeSurfer and FastCSR are also similar, with only 2.16% of the vertices showing significant difference (two-tailed paired *t*-tests, *p* < 0.01, i.e., − log_10_(*p*) > 2.0, FDR corrected). **c** The positive values in the sulcal depth maps indicate sulci (warm colors) and negative values indicate gyri (cool colors). For the ABIDE-II dataset, 2.44% of the vertices showed significant differences between the FreeSurfer and FastCSR method. Differences were mainly distributed in the insular cortices, the precentral gyrus, and the medial orbitofrontal cortices. **d** For the HCP dataset, 5.87% of the vertices showed significant difference in sulcal depth between FreeSurfer and FastCSR

In the first test, we compared cortical thickness and sulcal depth derived from FastCSR with those derived from the FreeSurfer pipeline. We observed high concordance between results obtained from these two different pipelines (Fig. 6). Specifically, for the ABIDE-II dataset, 0.07% of the vertices differed significantly with respect to cortical thickness (two-tailed paired *t*-tests, *p* < 0.01, FDR corrected) and 2.44% of the vertices differed significantly in sulcal depth (two-tailed paired *t*-tests, *p* < 0.01, FDR corrected). These differences were mainly observed in the insula, the precentral gyrus, and the OFC. Similarly, for the HCP dataset, 2.16% of the vertices differed significantly in cortical thickness (two-tailed paired *t*-tests, *p* < 0.01, FDR corrected) and 5.87% of the vertices were significantly different in regard to sulcal depth (two-tailed paired *t*-tests, *p* < 0.01, FDR corrected).

**Fig. 7:**
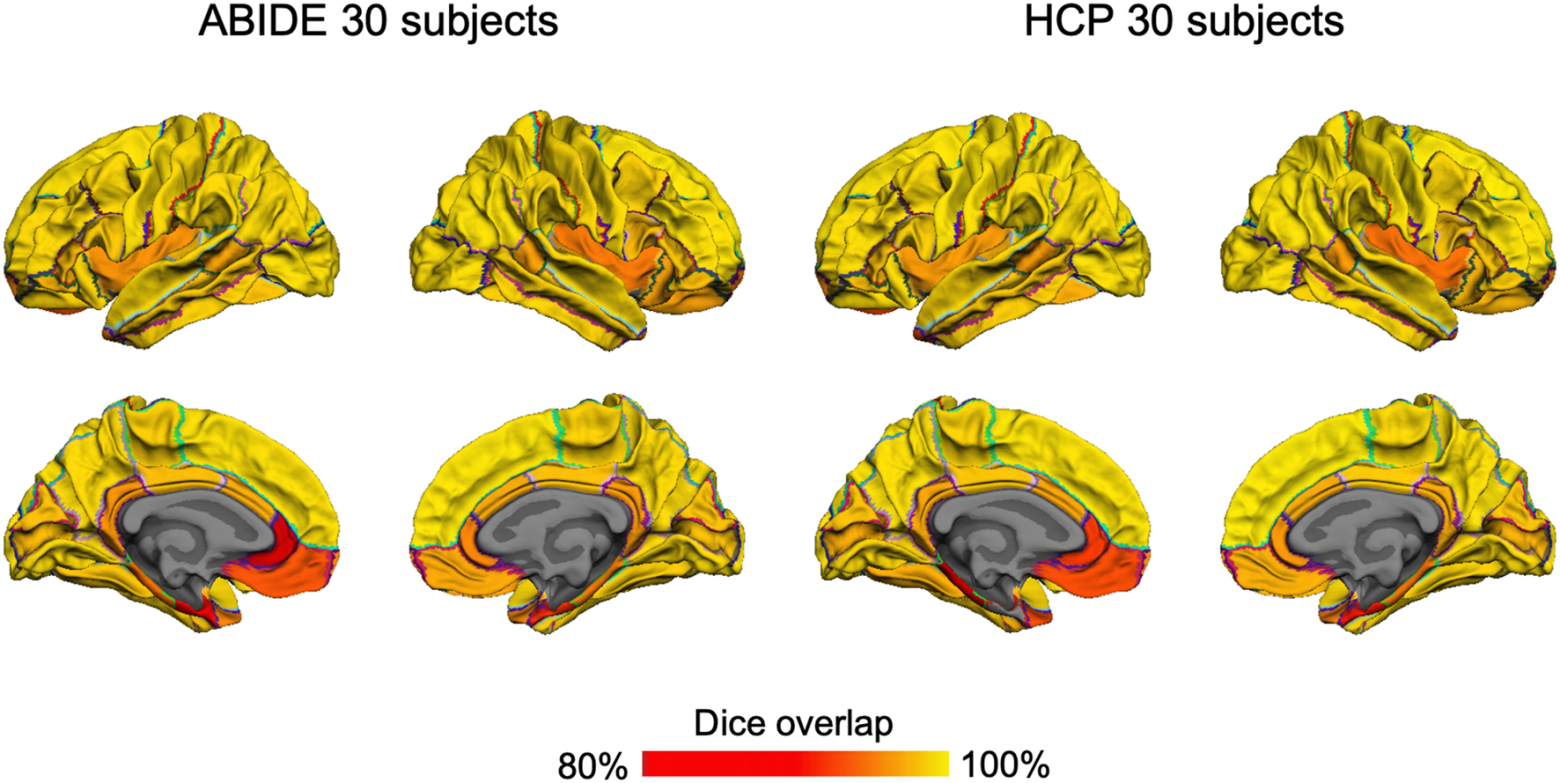
Anatomical parcellation in the ASD and HCP datasets. We assessed the similarity in anatomical cortical parcellation, measured by the Dice coefficient, for each cortical region obtained with FastCSR compared to FreeSurfer in both the ABIDE-II (left) and HCP (right) datasets. The Dice coefficients for most cortical areas (77.94% in ABIDE and 76.47% in HCP) are above 95%. Cortical areas with Dice coefficients smaller than 90% included the entorhinal region and the left rostral anterior cingulate cortex in both datasets, and the in the HCP dataset, additionally included the left parahippocampal area

In the second test, we assessed the anatomical parcellations in the unseen datasets. We employed the FreeSurfer automatic parcellation approach and segmented brain surfaces reconstructed by FastCSR into 34 anatomical regions. Similarity of anatomical parcellations derived from FastCSR and FreeSurfer pipelines was measured using the Dice coefficient. For the ABIDE-II dataset, the Dice coefficients ranged from 0.843 to 0.988. Moreover, 95.59% of parcels showed a Dice coefficient greater than 0.90 (Fig. 7, Additional file 1: Table S2), indicating high concordance in anatomical parcellations between these two pipelines. For the HCP dataset, the Dice coefficients range from 0.781 to 0.990. Similarly, 94.12% of parcels showed a Dice coefficient greater than 0.90 (Fig. 7, Additional file 1: Table S2). Taken together, these tests indicate that FastCSR can be generalized to unseen datasets, producing results comparable to that obtained with FreeSurfer, a pipeline that does not rely on training data.

### FastCSR showed high intra-subject test–retest reliability

**Fig. 8:**
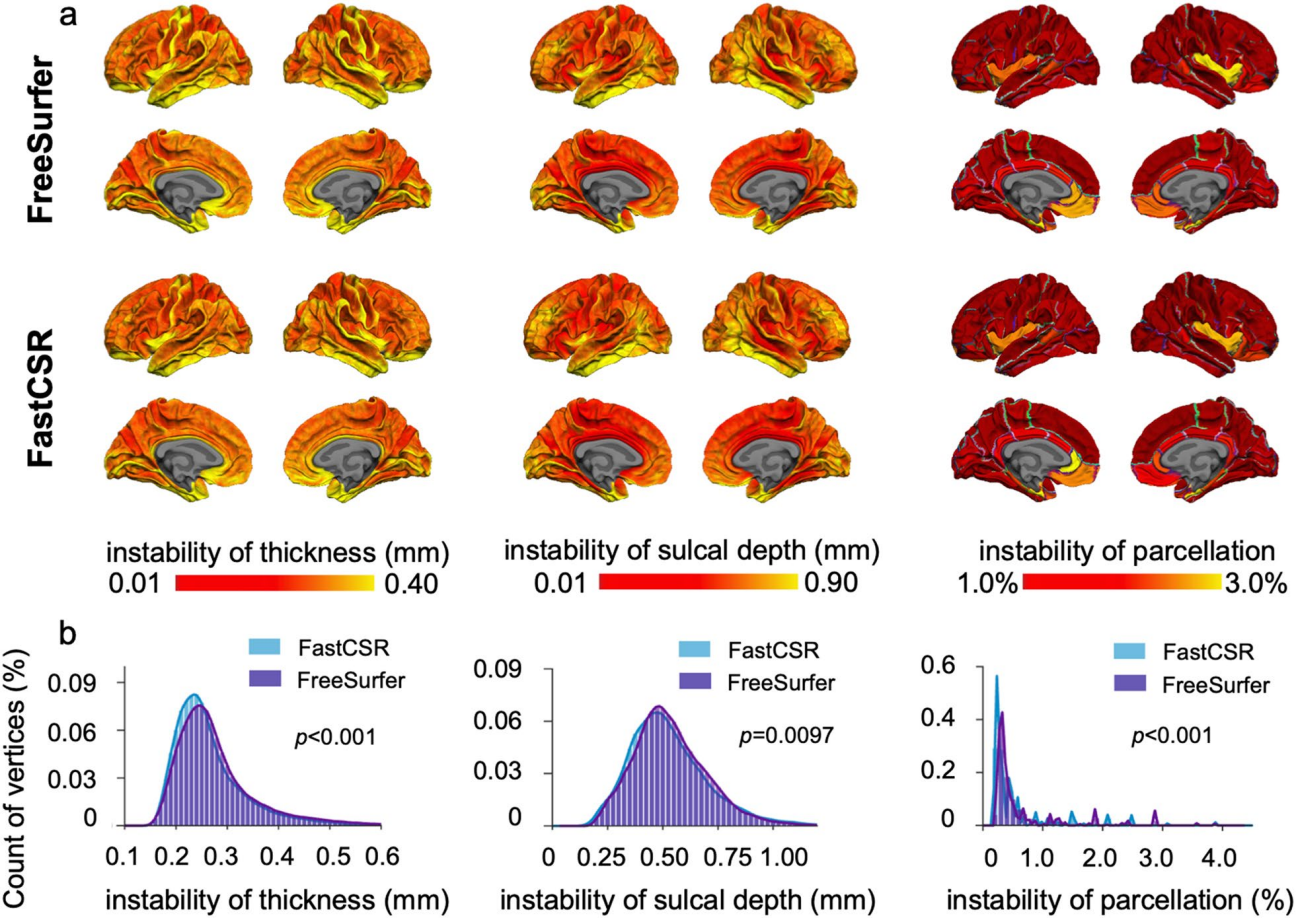
Surface morphometries and anatomical parcellation from FastCSR showed high intra-subject test–retest reliability. To examine the reliability of our FastCSR method, we measured the instability of surface morphometries and anatomical parcellation in a dataset consisting of 30 participants with 10 repeated scans for each participant. **A** The instability of morphometrics and parcellations was estimated by the standard deviation across the 10 sessions in each vertex for each participant. The lower instability, indicated by red color, suggests higher test–retest reliability. The average instability map across 30 individuals showed similar distributions for both the FreeSurfer (the upper panel) and FastCSR (the lower panel) methods. However, the FastCSR show lower instability for cortical thickness, sulcal depth, and parcellation than FreeSurfer. **B** Histograms illustrate the distribution of measurements obtained from FastCSR (blue bars) and FreeSurfer (purple bars). FastCSR shows lower instability relative to FreeSurfer in measures of cortical thickness (two-sample Kolmogorov–Smirnov test, *p* = 1.130 × 10^–7^), sulcal depth (two-sample Kolmogorov–Smirnov test, *p* = 9.700 × 10^–3^), and anatomical parcellation (two-sample Kolmogorov–Smirnov test, *p* = 1.037 × 10^–37^)

To assess the intra-subject test–retest reliability of results derived from FastCSR, we compared cortical thickness, sulcal depth and anatomical parcellation across different T1w scans of the same individual. We employed the CoRR-HNU dataset, in which each subject underwent 10 T1w scan sessions. Results derived from our FastCSR pipeline showed statistically greater test–retest reliability than that obtained from the FreeSurfer pipeline, including greater stability in cortical thickness (two-sample Kolmogorov–Smirnov test, *p* = 1.13 × 10^–7^), sulcal depth (two-sample Kolmogorov–Smirnov test, *p* = 9.70 × 10^–3^), and anatomical parcellation measures (two-sample Kolmogorov–Smirnov test, *p* = 1.04 × 10^–37^) (Fig. 8).

### FastCSR is robust for images of lower quality or with distortions

**Fig. 9:**
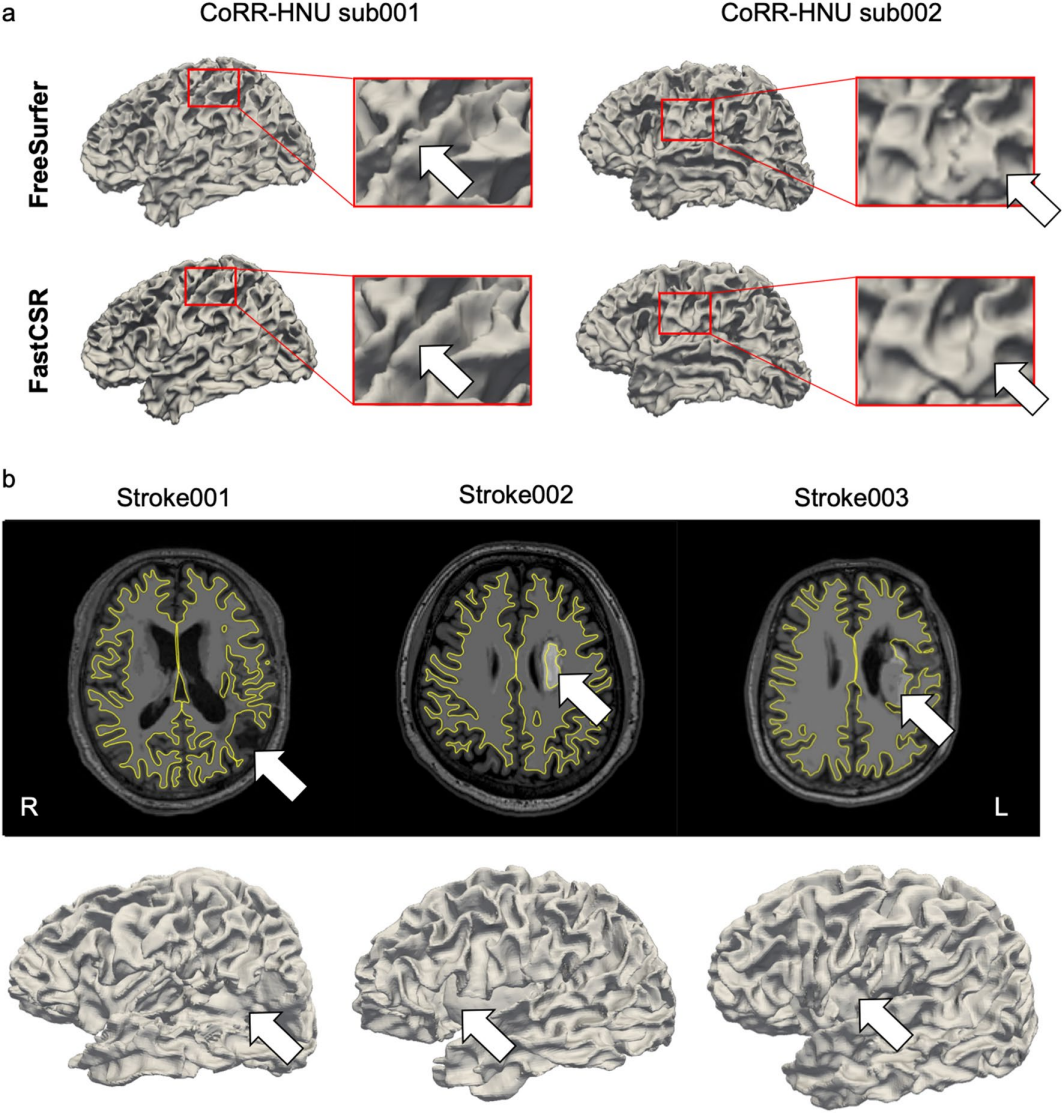
FastCSR is robust against image quality and brain distortions. **a** Cortical surfaces of two individuals with poor imaging quality from the CoRR-HNU dataset are reconstructed by FreeSurfer and FastCSR. White arrows highlight the jagged gyri caused by noise in the image (see upper panel) using the FreeSurfer pipeline. Surfaces obtained from the same individuals are reconstructed using our FastCSR method (see lower panel) and yielded cortical surfaces with smoother gyri. **b** FreeSurfer failed to reconstruct brain surfaces for three stroke patients whose brains are distorted due to lesions, whereas our FastCSR successfully reconstructed the cortical surfaces in these patients. The anatomical boundaries demarcating white matter and pial boundaries (yellow lines) overlaid onto horizontal sections of the T1w images with white arrows indicating the stroke lesions (upper panel). The corresponding cortical surfaces of the lesional hemispheres are displayed (lower panel). The white arrows indicate the stroke lesions

To test the robustness of FastCSR, we qualitatively checked the performance of the pipeline using a few subjects with lower data quality or with distortions. In a few individuals from the CoRR-HNU dataset that showed lower data quality, FreeSurfer produced jagged surface reconstructions near the pre- and post-central gyri, whereas FastCSR reconstructed these anomalous regions with smoother surfaces (see Fig. 9a for some examples), indicating that FastCSR might be more robust against poor image quality due to distortions.

Reconstructing surfaces for brains with distortions is challenging but critical when studying patients with stroke, trauma, or tumors [49–51]. While we trained the FastCSR model using normal healthy brains, we also tested the pipeline using three patients with obvious stroke lesions. Intriguingly, FastCSR successfully reconstructed their cortical surfaces while the FreeSurfer pipeline failed in all instances (Fig. 9b). Surface boundaries reconstructed by FastCSR depicted cortical folding details, as well as the geomorphological structure of the lesioned brain areas, suggesting that our FastCSR pipeline is relatively robust to images that contain distortions and performs superiorly to the FreeSurfer pipeline in patients with stroke-induced infarcts.

## Discussion

Here, we introduced a novel method for fast cortical surface reconstruction based on the 3D U-Net network architecture and the level set representation. Our results demonstrated that this method can efficiently reconstruct the cortical surface within five minutes, which is approximately 47 times faster than FreeSurfer and 7 times faster than FastSurfer. Brain surfaces reconstructed by our method showed comparable mesh quality with surfaces reconstructed using the FreeSurfer pipeline. As a method based on machine learning, our approach exhibited good generalizability in previously unseen datasets and showed high test–retest reliability. Finally, our method was robust to images of poor quality or with distortions. A fast, reliable method for cortical surface reconstruction has great potential in brain imaging research, as well as has important implications for clinical applications requiring fast and accurate surface renderings.

### FastCSR achieves high processing efficiency, mesh quality, and reliability

Our FastCSR approach aims to accelerate the automatization of CSR processing, while simultaneously remaining sensitive enough to capture fine-grained features of sulci and gyri and robustness in atypical cases wherein T1w images of individuals’ brains may be distorted due to lesions induced by cerebral infarct, and that often fail to be reconstructed by FreeSurfer. FreeSurfer is one of the most widely used pipelines with more than a 20-year history of processing individual anatomical images, including segmentation and automatic CSR [7]. Due to FreeSurfer’s high accuracy for performing CSR in typical brains, this approach has become the predominant method employed for reconstructing cortical surfaces with open-access datasets, such as the HCP, and other popular fMRI preprocessing pipelines, such as fMRIprep [17, 52]. However, despite its popularity, the low processing efficiency is the core bottleneck of FreeSurfer. To improve processing efficiency while simultaneously maintaining high accuracy, we trained a supervised learning model using level set representations of cortical surfaces reconstructed from FreeSurfer as the training labels. We elected to employ a supervised deep machine learning approach, given the high performance achieved in processing efficiency and accuracy when using this method, as well as its robustness in atypical cases wherein the brain is distorted due to trauma or injury.

Our FastCSR method significantly reduced the processing time compared to FreeSurfer while simultaneously preserving quality and reliability of the surface renderings, which may be explained by some advantages of the deep learning framework. First, surface-based topology correction within FreeSurfer represents a bottleneck from a computational perspective. This is because the processing time for this step is dependent upon the number of defective vertices on the original cortical surface, and because solution space grows exponentially as the number of defective vertices increases [53]. Although a genetic algorithm proposed by FreeSurfer accelerates the search for optimal solutions, the topology correction usually takes around 30 min depending on image quality. In contrast, FastCSR leverages the level set representation and the corresponding topology-preserving surface reconstruction to generate a cortical surface, significantly reducing computational costs [29, 54]. Second, to successfully and reliably reconstruct cortical surface, FreeSurfer and FastSurfer both require multiple preprocessing stages, including field uniformity normalization, nonlinear intensity normalization, skull stripping, and anatomical segmentation, that typically take hours to complete. In comparison, FastCSR only requires some very basic preprocessing steps such as including intensity normalization and white matter mask segmentation that can be finished within minutes.

Cortical surfaces reconstructed by FastCSR are comparable to results derived from FreeSurfer, as indicated by the surface displacement findings (Fig. 4) and quantitative measures of cortical morphometrics, such as cortical thickness, sulcal depth (Fig. 6) and anatomical parcellation (Fig. 7). Interestingly, FastCSR demonstrates higher test–retest reliability in morphometrics and anatomical parcellation across repeated scans of the same individuals compared to the FreeSurfer pipeline (Fig. 8). This suggests that the deep leaning model is able to learn some stable features from the training set that are collected from various sites with diverse noise distributions. Additionally, the data augmentation strategies used in the model training enriched the data diversity. In contrast, a traditional algorithm with little a priori knowledge about the population might be more vulnerable to image artifacts and produce less reliable results.

### Generalizability to unseen data

Compared with traditional computer vision algorithms, a common limitation of deep learning approaches is the uncertain generalizability to data outside the training dataset [26, 55]. To address this, we diversified our training set by incorporating various datasets that were acquired by different scanners and that differed in data quality and demographic distributions. Moreover, we leveraged a sliding-window strategy to split input images to multiple subunits [32, 56]. This strategy allows our model to flexibly receive input images with different sizes, capture local contextures more precisely, and reduce the model size [56]. We tested our FastCSR pipeline in unseen datasets with differing demographical distributions and scanning protocols. Our method showed good generalizability in these new datasets. Moreover, while our training set only included healthy participants, the model can reconstruct cortical surfaces for patients with brain disorders (i.e., the ABIDE-II dataset) or with relatively large anatomical lesions (i.e., the Stroke dataset). Importantly, FastCSR can reconstruct high-resolution images with very little extra cost in processing time compared to images of lower resolution. The capability and efficiency of our method for reconstructing high-resolution images will be appreciated as more and more studies utilize ultra-high field (7 T) MRI [57–59].

### Research and clinical implications

Our fast, robust pipeline for cortical surface reconstruction will benefit both neuroscience studies and clinical applications. First, FastCSR may facilitate various surface-based multimodal neuroimaging analyses including data derived from MEG, EEG, anatomical and functional MRI [17–19, 60–62]. While surface-based analyses may generate more precise spatial localization than traditional volume-based analyses [9], this analytical technique takes much time. For example, the first step of a widely used fMRI preprocessing pipeline, fMRIprep, is reconstructing cortical surfaces using FreeSurfer [17]. Combined with other time-consuming steps, preprocessing a single subject using fMRIprep takes several hours depending on the data quality and size, which limits its application in large-scale datasets. As more and more large-scale imaging studies are being conducted [10, 63, 64], computational resource costs have become an important consideration in data analyses. FastCSR can dramatically reduce the computational costs and accelerate analyses of data obtained from large-scale imaging studies while preserving data quality.

Importantly, FastCSR showed robustness to images with distortions, enabling surface-based analyses in many patients, such as those with trauma, stroke, or surgical resections, the most of which normally fail during the FreeSurfer cortical reconstruction step and require manual correction [49, 50]. The failures in FreeSurfer reconstruction in challenging cases exemplified by our stroke dataset may result from the relatively weak performance demonstrated by loss functions in the unsupervised learning algorithm. Given loss functions are highly dependent on a priori knowledge learned from datasets with typical brain morphometry and therefore, are particularly sensitive to abnormal image distributions. However, in the case of compromised images, this results in bad fitting in the original surface reconstruction and the generation of numerous topological surface defects. Moreover, the number of topological defects is often too large to correct within an acceptable time frame. Based on 3D U-Net architecture, our FastCSR approach extracts image features from local and global scales using overlapping convolutional kernels. During feature extraction, the local compromised features from brain lesions are subsequently smoothed. Thus, our FastCSR approach is capable of reconstructing cortical surfaces from distorted brains in a temporally efficient manner while simultaneously preserving surface topology. This method may facilitate future clinical applications that require fast and accurate surface processing and renderings, such as that required during intraoperative neuronavigation for tumor biopsy and resection.

### Limitations

The accuracy of FastCSR is dependent on the accuracy of labels in the training set. In this work, we directly employed the level set representations generated from FreeSurfer surfaces as labels to train our model. Hence, our network inevitably learned the systematic errors from FreeSurfer surfaces that were automatically reconstructed without manual correction. For example, in both the OFC and insular cortex, wherein image noise commonly affects local data quality, both FreeSurfer and FastCSR showed relatively low reliability. To further improve the accuracy of FastCSR in the future, a larger training dataset with manually corrected surfaces will be needed.

## Supplementary Information

**Additional file 1: Table S1.** Datasets summary of demographics, usages, sources, clinical states, and scanners. **Table S2.** Dice overlaps of anatomical parcellations between FastCSR and FreeSurfer.

## Data Availability

The datasets analyzed in the current study are available in the CoRR repository (http://fcon_1000.projects.nitrc.org/indi/CoRR/html/samples.html), the SALD repository (http://fcon_1000.projects.nitrc.org/indi/retro/sald.html), the HCP Young Adult repository (https://www.humanconnectome.org/study/hcp-young-adult), and the ABIDE II repository (http://fcon_1000.projects.nitrc.org/indi/abide/abide_II.html). The Stroke datasets analyzed during the current study are not publicly available due to privacy policies relating to clinical recordings but are available from the corresponding authors upon request. The source code and the pre-trained model of the FastCSR are available at GitHub: https://github.com/IndiLab/FastCSR.

## Abbreviations

MRI: Magnetic resonance imaging
FastCSR: Fast cortical surface reconstruction
3D: 3-Dimensional
CSR: Cortical surface reconstruction
T1w: T1-weighted
CoRR: The Consortium for Reliability and Reproducibility
SALD: The Southwest University Adult Lifespan Dataset
HNU: Hangzhou Normal University
HCP: Human Connectome Project
ABIDE-II: The Autism Brain Imaging Data Exchange II
IRB: Institutional Review Board
nnU-Net: No-new-U-Net
LReLU: Leaky rectified linear unit
MSE: The mean squared error
CV_PT_: The coefficient of variation in the processing time
ROI: Regions of interest
FDR: False discovery rate
OFC: The orbitofrontal cortex
ASD: Autism spectrum disorder
MEG: Magnetoencephalography
EEG: Electroencephalography
fMRI: Functional MRI
