# Supplementary material for: Fast cortical surface reconstruction from MRI using deep learning
Source: Brain Inform. 2022 Mar 9;9(1):6. doi: 10.1186/s40708-022-00155-7 (PMC8907118; doi:10.1186/s40708-022-00155-7)
Supplement: Supplementary file 1 — Additional file 1: Table S1. Datasets summary of demographics, usages, sources, clinical states, and scanners. Table S2. Dice overlaps of anatomical parcellations between FastCSR and FreeSurfer. [file 40708_2022_155_MOESM1_ESM.pdf]

Table 1. Datasets summary of demographics, usages, sources, clinical states, and scanners

Usage	No.	Name	Subjects	Age	Source	State	Scanner	1.5T/3T
Training & validation	1	CoRR_BNU	74	19-30	http://fcon_1000.projects.nitrc.org/indi/CoRR/html/bnu_1.html	normal	Siemens	3T
	2	CoRR_IACAS	27	19-43	http://fcon_1000.projects.nitrc.org/indi/CoRR/html/iacas_1.html	normal	GE	3T
	3	CoRR_IPCAS_1	25	18-24	http://fcon_1000.projects.nitrc.org/indi/CoRR/html/ipcas_1.html	normal	Siemens	3T
	4	CoRR_IPCAS_2	33	11-15	http://fcon_1000.projects.nitrc.org/indi/CoRR/html/ipcas_2.html	normal	Siemens	3T
	5	CoRR_IPCAS_3	29	17-25	http://fcon_1000.projects.nitrc.org/indi/CoRR/html/ipcas_3.html	normal	Siemens	3T
	6	CoRR_IPCAS_4	18	21-28	http://fcon_1000.projects.nitrc.org/indi/CoRR/html/ipcas_4.html	normal	GE	3T
	7	CoRR_IPCAS_5	22	18-19	http://fcon_1000.projects.nitrc.org/indi/CoRR/html/ipcas_5.html	normal	Siemens	3T
	8	CoRR_IPCAS_6	2	21-25	http://fcon_1000.projects.nitrc.org/indi/CoRR/html/ipcas_6.html	normal	Siemens	3T
	9	CoRR_IPCAS_7	67	6-17	http://fcon_1000.projects.nitrc.org/indi/CoRR/html/ipcas_7.html	normal	Siemens	3T
	10	CoRR_IPCAS_8	13	50-62	http://fcon_1000.projects.nitrc.org/indi/CoRR/html/ipcas_8.html	normal	Siemens	3T
	11	CoRR_XHCUMS	21	36-62	http://fcon_1000.projects.nitrc.org/indi/CoRR/html/xhcums_1.html	normal	Siemens	3T
	12	SALD	477	19-80	http://fcon_1000.projects.nitrc.org/indi/retro/sald.html	normal	Siemens	3T
Processing time	13	A subset of the validation dataset	30	/	/	normal	GE, Siemens	3T
Test-retest reliability	14	CoRR_HNU	30	20-30	http://fcon_1000.projects.nitrc.org/indi/CoRR/html/hnu_1.html	normal	GE	3T
Distorted brain	15	Stroke	9	37-71	In-house	stroke	Philip	3T
Generalizability	16	ABIDE II	30	5-64	http://fcon_1000.projects.nitrc.org/indi/abide/abide_II.html	ASD	Philips, GE, Siemens	1.5T/3T
	17	HCP	30	22-35	https://www.humanconnectome.org/study/hcp-young-adult	normal	Siemens	3T

Table S2. Dice overlaps of anatomical parcellations between FastCSR and FreeSurfer

Parcellated structure name	ABIDE 30 subjects		HCP 30 subjects	
	LH Dice overlap (mean ± std.)	RH Dice overlap (mean ± std.)	LH Dice overlap (mean ± std.)	RH Dice overlap (mean ± std.)
bankssts	0.953 ± 0.014	0.950 ± 0.011	0.947 ± 0.020	0.947 ± 0.019
caudalanteriorcingulate	0.958 ± 0.012	0.957 ± 0.013	0.954 ± 0.018	0.958 ± 0.012
caudalmiddlefrontal	0.980 ± 0.003	0.976 ± 0.004	0.982 ± 0.004	0.979 ± 0.005
cuneus	0.967 ± 0.007	0.969 ± 0.007	0.969 ± 0.008	0.970 ± 0.009
entorhinal	0.862 ± 0.074	0.892 ± 0.042	0.781 ± 0.054	0.876 ± 0.066
fusiform	0.969 ± 0.008	0.976 ± 0.006	0.958 ± 0.008	0.978 ± 0.005
inferiorparietal	0.982 ± 0.003	0.985 ± 0.002	0.983 ± 0.003	0.986 ± 0.003
inferiortemporal	0.976 ± 0.005	0.978 ± 0.005	0.975 ± 0.005	0.982 ± 0.003
isthmuscingulate	0.944 ± 0.014	0.947 ± 0.013	0.955 ± 0.008	0.958 ± 0.010
lateraloccipital	0.984 ± 0.002	0.983 ± 0.003	0.984 ± 0.004	0.984 ± 0.004
lateralorbitofrontal	0.978 ± 0.004	0.952 ± 0.022	0.982 ± 0.003	0.963 ± 0.027
lingual	0.982 ± 0.004	0.981 ± 0.004	0.980 ± 0.006	0.981 ± 0.005
medialorbitofrontal	0.909 ± 0.032	0.955 ± 0.015	0.905 ± 0.028	0.960 ± 0.011
middletemporal	0.973 ± 0.007	0.976 ± 0.004	0.972 ± 0.005	0.979 ± 0.004
parahippocampal	0.923 ± 0.040	0.932 ± 0.030	0.834 ± 0.112	0.929 ± 0.046
paracentral	0.975 ± 0.006	0.977 ± 0.005	0.978 ± 0.006	0.981 ± 0.003
parsopercularis	0.972 ± 0.004	0.965 ± 0.008	0.975 ± 0.005	0.969 ± 0.006
parsorbitalis	0.961 ± 0.009	0.966 ± 0.009	0.967 ± 0.007	0.973 ± 0.005
parstriangularis	0.965 ± 0.007	0.971 ± 0.006	0.969 ± 0.006	0.972 ± 0.005
pericalcarine	0.965 ± 0.011	0.969 ± 0.008	0.957 ± 0.014	0.961 ± 0.019
postcentral	0.981 ± 0.003	0.980 ± 0.004	0.985 ± 0.003	0.985 ± 0.002
posteriorcingulate	0.966 ± 0.007	0.968 ± 0.009	0.971 ± 0.007	0.974 ± 0.006
precentral	0.984 ± 0.002	0.982 ± 0.002	0.987 ± 0.002	0.986 ± 0.002
precuneus	0.981 ± 0.004	0.982 ± 0.003	0.985 ± 0.003	0.985 ± 0.002
rostralanteriorcingulate	0.843 ± 0.095	0.936 ± 0.021	0.894 ± 0.077	0.946 ± 0.018
rostralmiddlefrontal	0.980 ± 0.003	0.985 ± 0.003	0.983 ± 0.004	0.986 ± 0.002
superiorfrontal	0.984 ± 0.003	0.988 ± 0.002	0.987 ± 0.003	0.990 ± 0.001
superiorparietal	0.983 ± 0.003	0.983 ± 0.002	0.987 ± 0.002	0.985 ± 0.003
superiortemporal	0.980 ± 0.005	0.980 ± 0.004	0.979 ± 0.008	0.980 ± 0.007
supramarginal	0.980 ± 0.003	0.981 ± 0.004	0.983 ± 0.003	0.982 ± 0.005
frontalpole	0.937 ± 0.021	0.931 ± 0.017	0.942 ± 0.016	0.945 ± 0.016
temporalpole	0.936 ± 0.023	0.939 ± 0.023	0.904 ± 0.031	0.935 ± 0.019
transversetemporal	0.965 ± 0.008	0.952 ± 0.016	0.959 ± 0.027	0.948 ± 0.032
insula	0.944 ± 0.022	0.930 ± 0.038	0.948 ± 0.018	0.925 ± 0.023

Note: LH = Left Hemisphere; RH = Right Hemisphere
